# The Relationship between Moral Sensitivity and Professional Behaviour and Its Comparison in First- and Last-Year Undergraduate Nursing Students

**DOI:** 10.1155/2023/5368045

**Published:** 2023-11-23

**Authors:** Maryam Bagheri, Mohsen Shahriari, Pegah Hassanvand, Akram Mohammadi Pelarti, Afarin Ghanavatpour

**Affiliations:** ^1^Nursing and Midwifery Care Research Center, Department of Adult Health Nursing, School of Nursing and Midwifery, Isfahan University of Medical Sciences, Isfahan, Iran; ^2^Department of Adult Health Nursing, School of Nursing and Midwifery, Isfahan University of Medical Sciences, Isfahan, Iran; ^3^Shafa Specialized Hospital, Isfahan University of Medical Sciences, Isfahan, Iran; ^4^Shariati Hospital, Isfahan, Iran

## Abstract

**Background:**

Nursing students should be equipped with ethical sensitivity and professional behaviour because they will face challenging ethical issues in their future work environment. This study aimed to determine the relationship between moral sensitivity and professional behaviour and compare it in first- and last-year undergraduate nursing students.

**Methods:**

This study was a cross-sectional, correlational study that was conducted at Isfahan University of Medical Sciences in 2019. The sample size of this study was 238 nursing students. The tools used in this study were the Persian versions of the moral sensitivity questionnaire and the professional behaviour questionnaire. The data were analyzed using SPSS 18 software.

**Results:**

Linear regression showed that the total score of moral sensitivity of nursing students had a significant relationship with their professional behaviour (*p*  <  0.05). The result of the univariate analysis showed that the mean total score of moral sensitivity and professional behaviour was significantly higher in the last year than in first-year students (*p*  <  0.05).

**Conclusion:**

Considering the relationship between moral sensitivity and the professional behaviour of nursing students, the promotion of moral sensitivity can become the basis for the development of the professional behaviour of nursing students. Therefore, it is suggested to focus on teaching the principles of nursing ethics to develop the moral sensitivity of undergraduate nursing students.

## 1. Introduction

Nurses have an essential role in upgrading the care and health of patients which depends on the quality of performance and professional behaviour of nurses [1]. In this regard, promoting the quality of the nursing services provided to patients requires nurses to employ scientific care and moral principles in their professional practice [2, 3]. Ethics is a pillar in nursing practice, and a critical part of the nursing profession is to achieve moral competency in parallel with clinical competency [4]. Nurses are responsible for making the best professional and ethical care decisions, which necessitates moral sensitivity and professional behaviour [3, 5, 6].

Moral sensitivity is a crucial component of ethical decision-making, which refers to the ability that involves recognizing the ethical dilemmas and their effects, the vulnerability of patients, and comprehending the implications of ethical decision-making [7, 8]. The lack of moral sensitivity makes it impossible for a person to recognize nursing ethical problems [9]. Nursing students, as future healthcare providers, should be equipped with advanced degrees of moral sensitivity because they will face challenging ethical issues in their future work environment [10, 11]. Nonetheless, most studies show that nursing students possess different levels of moral sensitivity [12–14]. In a study by Akca et al. [14], it was specified that nursing students had moderate levels of moral sensitivity [14]. Nevertheless, a study by Ozcetin and Hicdurmaz showed that the moral sensitivity score decreased with increasing age and educational level, and the students who passed ethics courses obtained lower scores in some subscales of moral sensitivity, as well as regarding the total score [15]. In addition to paying attention to ethical issues in the structure of nursing education curricula, attention should be paid to the development of innovative content and educational methods at the undergraduate level [11, 13]. However, the effectiveness of ethics courses and proper educational strategies in nursing is controversial [16–19].

Professional behaviour is among the fundamental concepts of the nursing profession; so, inappropriate professional performance can lead to job burnout, reduction or loss of motivation, and demotion of job satisfaction among nurses [20]. Similarly, nursing students' professional values are determined by their professional behaviours and performance [21]. However, there has been debate regarding professionalism and the acquisition of skills in nursing, so that in some studies asserting that newly graduated nurses lack the readiness required for fulfilling complex and professional nursing practices, including clinical skills, logical thinking, time management, communication skills, and teamwork skills [22, 23]. In a study by Nabavi et al. [24], it was reported that although nursing students attained desirable levels of professionalism during their first years of academic education, they not only failed to progress in this area but also experienced a downturn during the final years of education [24].

Higher levels of moral sensitivity in nursing students can be related to their greater professional commitments [25]. Chen et al. [8] showed that there was a positive correlation between moral sensitivity and professional values, and professional values played a mediating role in the relationship between moral sensitivity and ethical decision-making [8]. Baykara et al. [26] study found that moral sensitivity has a positive effect on professionalism; therefore, it can be said that while moral sensitivity is a prerequisite for the development of professionalism, professionalism also accelerates the development of moral sensitivity [26].

Undergraduate nursing educational program in Iran for teaching ethical principles includes a course called “nursing ethics and professional communication” (1.5 credits and a total of 34 hours) [27].

Even though nursing students should recognize ethical challenges and overcome them by showing professional behaviour and making ethical decisions, very limited studies have been conducted in the field of evaluating the relationship between ethical sensitivity and the professional behaviour of nursing students. Also, there are inconsistencies and uncertainties regarding the effectiveness of ethics education in terms of moral sensitivity and professional behaviour during the undergraduate nursing course. In this regard, the investigation of moral sensitivity and professional behaviour, as essential components of the development of professional ethics, can elucidate the effectiveness of ethics education during the undergraduate nursing course. Therefore, the present study aimed to determine the relationship between moral sensitivity and professional behaviour and compare them between first- and last-year nursing students.

## 2. Materials and Methods

### 2.1. Study Design

This was a *cross*-*sectional, correlational study* to investigate the relationship between ethical sensitivity and professional behaviour, and also, this study compares moral sensitivity and professional behaviour between the first- and last-year nursing students of Isfahan University of Medical Sciences in 2019.

### 2.2. Participants and Sampling

In the second semester of 2019, 289 students were registered in the first year and the last year. The inclusion criteria for this study included being first-year and last-year nursing students, not having a critical incident in the last 6 months, and not suffering from mental disorders. The exclusion criteria from this study included unwillingness to continue participating in the study and incomplete questionnaires. Sampling was conducted by census method. Of 289 students who were registered in the semester, 238 nursing students, including 158 first-year students and 80 final-year students, completed questionnaires.

### 2.3. Instruments

A three-part questionnaire was used to collect the data. The first part of the questionnaire addressed demographic characteristics. The second part of the questionnaire included the Persian version of Lutzen's moral sensitivity (1994) scale in determining the subjects' moral sensitivity, whose validity and reliability were confirmed by Hasanpoor et al. [28]. The original version of this questionnaire was made by Lutzen and Nordin [29]. This questionnaire contained 25 queries organized into six subdomains, including respecting the patient's autonomy, knowing how to communicate with the patient, professional knowledge, ethical conflicts experienced, applying moral concepts during ethical decision-making, and honesty and benevolence. These queries were scored on a 5-point Likert scale from totally disagree to totally agree. The minimum and maximum of the total score were 0 and 100, respectively. Hasanpoor et al. assessed the reliability of the questionnaire using Cronbach's alpha method, reporting a coefficient of 81% [28].

The third part of the data collection tool included the Persian version of the Goz professional behaviour (2010) questionnaire, which is in the study by Nabavi et al. [24]; The original version of this questionnaire was made by Goz and Geckil [30]. The validity of this questionnaire was approved by experts in the field, and its reliability using Cronbach's alpha method was reported as above 0.70 [24]. This scale includes 27 items that were scored between 1 and 5, delivering a total score ranging from 27 to 135 [24].

### 2.4. Data Collection

All the data were collected by only one of the researchers during 1 month (15 May to 15 June) in the second semester of 2019. Data collection was carried out from first-year students in the nursing faculty and last-year students in the hospital in the morning (the questionnaires were completed during students' break). After stating the objectives of the research and obtaining informed consent from the students, the questionnaires were given to the students and the students completed them in a self-report form. The duration of completing the questionnaires by the participants was between 15 and 20 minutes.

### 2.5. Ethical Considerations

This study was approved by the Ethics Committee of Isfahan University of Medical Sciences (the code of IR.MUI.RESEARCH.REC.1398.200). Sample recruitment was started after the study approval, getting the scientific code, and necessary coordination was made with the research deputy of the university after elaborating the objectives and procedures of the study. Eligible students have explained the objectives of the study and the instructions for completing the questionnaire. Written informed consent was obtained from all participants. Participation in the study was completely voluntary, and the individuals' data remained entirely confidential.

### 2.6. Analytical Methods

The data were analyzed using SPSS 18 software. The results of qualitative variables were reported as numbers (percentage), and quantitative variables were reported as mean ± standard deviation. To compare the subscales of moral sensitivity based on demographic variables, multivariate analysis was used, and to compare the total score of moral sensitivity and professional behaviour, independent univariate analyses and one-way analysis of variance were used. To investigate the effect of subscales of moral sensitivity (on the feeling of standard score) and its total score taking into account the side variables on students' professional behaviour, multiple and simple regressions were used. The results were reported at a significance level of 0.05.

## 3. Results

The results showed that out of the participants, 158 were first-year students. The mean age was 20.34 ± 3.01 years, and the mean GPA was 16.93 ± 2.11. Most of the participants were single and lived with their families. There were 80 last-year students. Their mean age was 23.31 ± 1.87 years, and their mean GPA was 16.30 ± 1.06. Most of the last-year students were single and lived in the dormitory (Table 1).

The results of univariate and multivariate analyses showed that there was no significant difference between the total score of moral sensitivity and the score of its subscales and the score of students' professional behaviour based on marital status, housing, apprenticeship, working, and having another degree (*p*  >  0.05).

The results of multivariate analysis showed that there was not a significant difference between the mean score of the moral sensitivity subscales of men and women (*p* = 0.357), but the mean total score of moral sensitivity and professional behaviour of women was significantly higher than men (*p*  <  0.05).

The significance of the results of multivariate analysis showed that there was a significant difference in at least one of the subscales of moral sensitivity in first- and last-year nursing students (*p* = 0.002) so, based on univariate analysis, it was found that the mean of all subscales of moral sensitivity and professional behaviour in last-year students was significantly higher than first-year students (*p*  <  0.05).

Pearson's linear correlation between grade point average and age of students with professional behaviour scores was equal to (*r* = −0.03, *p* = 0.718) and (*r* = 0.02, *p* = 0.815), respectively, and with moral sensitivity score was equal to (*r* = −0.11, *p* = 0.167) and (*r* = 0.20, *p* = 0.003), respectively, which indicated a weak positive linear correlation between age and the total score of moral sensitivity that was significant. In other cases, this correlation was not significant, and the correlation between professional behaviour and the total score of moral sensitivity was *p*  <  0.001, *r* = 0.39, which indicated a positive (moderate) and significant correlation between the two variables (Table 2).

To investigate the effect of moral sensitivity subscales on students' professional behaviour scores by using multiple regression and observing their standardized coefficients, it was determined that the “Professional Knowledge” subscale had the most significant positive effect on the mean score of students' professional behaviour (*p*  <  0.001). The subscale of “Honesty and Benevolence” also had an insignificant effect but considerable (*p* = 0.055). The effect of other subscales on professional behaviour was not significant. A similar result was obtained after adjusting for the group effect (Table 3).

**Figure 1:**
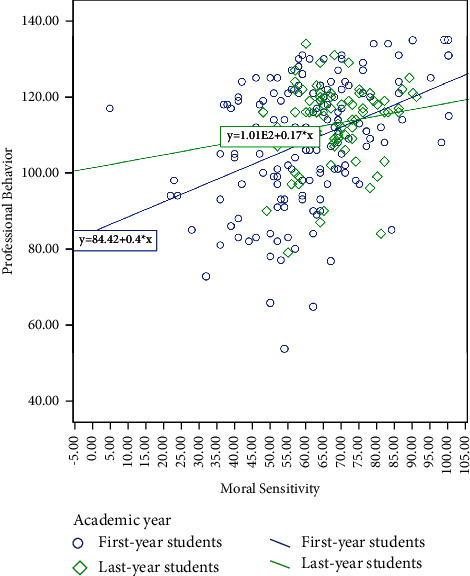
The scatter plot of the moral sensitivity score against professional behaviour for first- and last-year nursing students.

For assessing the effect of students' moral sensitivity on their professional behaviour with regard to students' academic year, first, a separate simple linear regression was fitted in the group of first-year and last-year students. The results showed that in the first-year students, this relationship was not significant, and in the last-year students, this relationship was significant, which indicated the interaction effect between academic year and moral sensitivity. For this purpose, the scatter plot of the score of moral sensitivity against the professional behaviour of students was drawn simultaneously for first- and last-year students, which is shown in Figure 1. As it can be seen, this point of intersection occurred almost at the score of 75 moral sensitivity, which showed that the score of moral sensitivity can be divided into two intervals (<75 and ≥75) (it showed the same pattern in the score below 75), and this is the cut point indicates high moral sensitivity [28, 29] (Figure 1).

The regression model of the effect of the academic year (first- and last-year) and moral sensitivity score (<75 and ≥75) and their simultaneous effect were fitted once in a simple way and once by adjusting the effect of gender on the professional behaviour score of students (Table 4). The results showed that in both models, the interaction effect of the academic year on moral sensitivity was significant. Assuming that the effect of gender is constant, the results of the modified model showed that the professional behaviour score of last-year students with a moral sensitivity score above 75 was 5.91 points lower on average compared to first-year students. Also, the score of professional behaviour in last-year students with a sensitivity score of 75> was higher by 6.94 points on average compared to first-year students.

## 4. Discussion

The study aimed to investigate the relationship between moral sensitivity and professional behaviour of nursing students and compare them between first-year and last-year students. The results of our study indicated the existence of a positive and direct relationship between the level of moral sensitivity and professional behaviour of nursing students. Some studies showed that promoting moral sensitivity in nursing students may lead to their professional values or attitudes development [8, 25, 26]. Compliance with ethical principles is one of the criteria required for professionalism in nursing [31]. The results of Chen et al. [8] also indicated the existence of a positive correlation between moral sensitivity, professional values, and moral decision-making of nursing students [8]. According to previous studies, professional behaviour is a fundamental concept in the nursing profession [20] that can play an important role in clarifying the impact of ethics education on becoming professional and acquiring moral qualifications in the course of students' studies [8, 26, 32]. In this regard, education can be an important element in acquiring professional attitudes and developing the moral sensitivity of nursing students [26].

The results of the present study showed that women students attained significantly higher total scores of moral sensitivity and professional behaviour compared to men. In previous studies, different results were presented on the effect of gender on the level of moral sensitivity [14, 15, 33–35]. In some other studies, the level of moral sensitivity of female nursing students was higher than that of males [33, 34]. In contrast with the present study, some studies have found no significant relationship between the moral sensitivity score and nursing students' gender [14, 15, 35].

According to several studies, age can also affect moral sensitivity, and some studies have noted a positive correlation between age and the moral sensitivity score [36, 37]. However, in other studies such as the study of Borhani et al. [33], no significant relationship between age and moral sensitivity was reported among nurses [33]. In the present study, since last-year students had significantly higher mean age and mean score of moral sensitivity compared to their first-year counterparts, it can be argued that increasing age and, therefore, achieving more mental maturity can gradually improve students' moral sensitivity [36].

The results indicated that last-year nursing students had significantly higher levels of moral sensitivity compared to first-year students. Considering that undergraduate nursing students pass a theory course on nursing ethics and attend longer times in clinical environments in their last educational year, last-year students are expected to have higher moral sensitivity than first-year students. In addition, during the last year, nursing students integrate their clinical reasoning skills with ethics at the highest level, and they have maximum maturity [11]. Kohansal et al. [38] reported that the mean score of moral sensitivity was higher in students studying in the eighth academic semester compared to those in the third semester, indicating a significant link between moral sensitivity and the academic semester [38] which is aligned with the results of our study regarding the effect of the academic year on moral sensitivity. However, Baykara et al. [26] declared that students' moral sensitivity decreased with elevating educational years [15, 26]. In another study, Tuvesson et al. [34] also identified no evidence indicating the improvement of moral sensitivity by passing academic years [34]. The inconsistency between these results may be related to the factors affecting training, such as the classroom environment, educational traits, level of preparedness, and the research tools used in studies [6]. Consequently, it is necessary to pay attention to the significant role of education and the use of effective ethical teaching strategies to upgrade students' moral sensitivity [38].

In the present study, both groups of students attained the highest score in the domain of “professional knowledge” and the lowest score in the domain of “honesty & benevolence.” In contrast with this study, Mostafavian et al. [35] described that the highest score obtained by students was related to the domain of “knowing how to communicate with the patient” [35]. In the study of Kohansal et al. [38], the highest mean score was related to the domain of “honesty & benevolence” while the lowest scores were obtained in the domains of “professional knowledge” and “applying moral principles when making ethical decisions” [38], which opposed the findings of the present study. This inconsistency could be related to the different student populations, so that, in the present study, last-year students were compared with first-year students who had limited clinical experience. Likewise, in the study of Borhani et al. [36], who enrolled undergraduate nursing students, the domains of “expressing benevolence” attained the highest mean scores, this domain refers to concepts such as honesty, trust between the nurse and the patient, considering patients' reactions to care, and patients' perception and knowledge about their disease [36]. Establishing a suitable and sympathetic relationship with the patient along with honesty and benevolence can lay the ground for winning patients' trust [36, 38]. The fact that both groups of first-year and last-year students obtained the lowest scores in the “honesty and benevolence” dimension might reflect the weakness of students in dealing with ethical dilemmas and applying the theoretical ethical concepts learned in the clinical setting. This issue highlights the need for nursing students to receive more training during their education on how to apply professional ethics principles, beneficence, and nonmaleficence. Moral sensitivity is created through education, but it is established through professional competency and showing ethically acceptable professional behaviours [14].

In the present study, both groups of students enrolled had the desired levels of professional behaviour. Some studies have indicated that students possess desirable levels of professional behaviour [32, 39], and others, such as Taylan et al. [21], have reported moderate-level professional behaviour among nursing students [21]. The differences observed in students' levels of professional behaviour in various studies may be related to features such as the place of studying, as well as value and cultural differences among communities [39]. In addition, some demographic characteristics of students can influence their professional behaviour. In this regard, Nemati et al. [32] noticed that students' age and GPA were positively associated with their professional behaviour scores [32]. However, in the present study, the student's professional behaviour scores had no significant relationship with their GPA. The mean score of professional behaviour was significantly higher among last-year students compared to first-year students. Nevertheless, Nemati et al. stated that none of the professional skills of students were related to their academic semester [32].

In this study, by adjusting for gender, it was found that the professional behaviour score of last-year's students with high moral sensitivity was lower compared to first-year students. Because of some organizational and professional factors, nursing students may face problems in developing professional behaviours [21, 40]. Factors such as the existence of an inappropriate pattern in the clinical environment, conflict with other members of the healthcare team, nonstandard performance of nurses, and the influence of nurses and students on each other can be effective for this issue [21, 37]. The professional socialization process of last-year students may be destroyed during the process of transition to the clinical setting and becoming independent due to the gap between theory and practice [41]. Therefore, nursing students may be influenced by nurses' behavioural patterns or suppressive behaviours which lead to inappropriate professional behaviour, despite having high moral sensitivity.

## 5. Conclusions

Our study showed that there is a positive relationship between the moral sensitivity of nursing students and their professional behaviour. In addition, the results indicated that last-year nursing students had significantly higher levels of moral sensitivity and professional behaviour compared to their first-year students. This finding may denote that the educational content of nursing undergraduate courses and the more encounter of students with ethical conflicts in the clinical environment can influence their perception of ethical challenges and how to handle them. Nursing students are believed to struggle to learn professional behaviours during educational courses [21]. Therefore, it may be beneficial to use novel educational methods that acquaint students with real clinical situations, which may lead to achieving a correct understanding of the application of ethical concepts and conundrums in ethical decisions. The relationship between the moral sensitivity of nursing students and their professional behaviour can suggest an important role in explaining the effect of ethics education on professionalization and the acquisition of moral qualifications in the course.

## Figures and Tables

**Table 1:** Frequency distribution of demographic variables of nursing students.

Variables	Level	*n* (%)
Gender	Female	101 (42.4)
	Male	127 (53.4)

Marital status	Married	31 (13.6)
	Single	197 (86.4)

Housing	Dormitory	105 (45.9)
	Living with family	116 (50.7)
	Students house	8 (3.4)

Interest in the field	Yes	166 (69.7)
	No	56 (23.5)

Apprenticeship at the same time	Yes	31 (13.8)
	No	194 (86.1)

Working at the same time	Yes	25 (11.1)
	No	200 (88.91)

Another degree	Yes	19 (8.5)
	No	204 (91.5)

**Table 2:** Comparison of the total and subscale scores of moral sensitivity and professional behaviour in nursing students based on demographic variables.

Variables	Respect for autonomy	Professional knowledge	How to communicate	Ethical conflicts	Applying moral concepts	Honesty and benevolence	Total moral sensitivity	Professional behaviour
*Gender*								
Female	11.69 ± 4.14	15.15 ± 3.84	8.44 ± 2.4	12.61 ± 3.42	12.09 ± 4.2	5.06 ± 1.73	65.06 ± 15.36	111 ± 12.9
Male	11 ± 3.87	14.11 ± 4.2	7.82 ± 2.55	11.57 ± 3.61	11.25 ± 4.07	4.9 ± 1.81	60.68 ± 16.11	107 ± 16.69
*p* value	*P *_multivariate_ = 0.357						0.04	0.042

*Marital status*								
Married	11.25 ± 3.61	13.42 ± 4.61	8.55 ± 2.07	11.76 ± 3.19	11.39 ± 3.79	5.3 ± 1.31	61.69 ± 14.5	106.32 ± 17.87
Single	11.32 ± 4.05	14.75 ± 3.97	8 ± 2.55	12.05 ± 3.6	11.65 ± 4.19	4.94 ± 1.81	62.74 ± 16	109.86 ± 14.71
*p* value	*P *_multivariate_ = 0.122						0.736	0.229

*Housing*								
Dormitory	11.32 ± 3.72	14.15 ± 3.94	8.04 ± 2.5	12.25 ± 3.37	11.71 ± 3.82	4.81 ± 1.84	62.3 ± 14.94	108.06 ± 15.81
With family	11.35 ± 4.03	14.85 ± 4.23	8.17 ± 2.46	11.76 ± 3.69	11.44 ± 4.33	5.04 ± 1.69	62.64 ± 16.39	110.41 ± 14.49
Students house	10.43 ± 6.39	15.75 ± 3.15	7 ± 2.82	12.12 ± 3.75	13.18 ± 5.16	6.12 ± 1.8	64.6 ± 20.48	109.43 ± 17.75
*p* value	*P *_multivariate_ = 0.053						0.921	0.52

*Apprenticeship*								
Yes	11.3 ± 3.35	15 ± 3	7.85 ± 1.9	12.36 ± 2.73	12.04 ± 3.59	5.12 ± 1.7	63.7 ± 11.33	111.21 ± 17
No	11.28 ± 4.1	14.46 ± 4.23	8.07 ± 2.58	11.95 ± 3.68	11.56 ± 4.22	4.93 ± 1.78	62.29 ± 16.56	108.89 ± 15.04
*p* value	*P *_multivariate_ = 0.894						0.647	0.434

*Working*								
Yes	11.85 ± 3.67	13.91 ± 4.51	7.76 ± 2.14	12.66 ± 3.03	12.62 ± 3.43	5.48 ± 1.44	64.28 ± 14.44	105.16 ± 21.01
No	11.19 ± 4	14.58 ± 4.02	8.06 ± 2.52	11.88 ± 3.58	11.58 ± 4.09	4.9 ± 1.8	62.08 ± 15.89	109.56 ± 14.3
*p* value	*P *_multivariate_ = 0.182						0.512	0.317

*Another degree*								
Yes	11.65 ± 4.88	15.48 ± 4.16	8.76 ± 2.25	13.15 ± 3.45	12.76 ± 4.43	5.89 ± 1.28	67.72 ± 15.23	109.17 ± 15.18
No	11.19 ± 3.86	14.43 ± 4.09	8 ± 2.49	11.84 ± 3.51	11.49 ± 4.06	4.87 ± 1.8	61.84 ± 15.76	109.15 ± 15.29
*p* value	*P *_multivariate_ = 0.223						0.120	0.995

*Academic year*								
First year	10.7 ± 4.27	13.96 ± 4.48	7.8 ± 2.63	11.54 ± 3.84	11.18 ± 4.32	4.71 ± 1.81	59.91 ± 17.2	108.44 ± 16.68
Last year	12.71 ± 2.92	15.8 ± 2.64	8.62 ± 2.05	13.02 ± 2.51	12.55 ± 3.5	5.5 ± 1.54	68.23 ± 10.25	112.37 ± 11.35
*p* value	<0.001	0.001	0.017	0.002	0.016	0.001	<0.001	0.034

**Table 3:** Regression model of the effect of moral sensitivity subscales on the professional behaviour score of nursing students.

	Respect for autonomy	Professional knowledge	How to communicate	Ethical conflicts	Applying moral concepts	Honesty and benevolence
	Standardized coefficient (*p* value)					
Model I^*∗*^	0.065 (0.483)	0.352 (<0.001)	0.078 (0.308)	−0.059 (0.482)	−0.027 (0.773)	0.139 (0.055)
Model II^*∗∗*^	0.058 (0.532)	0.346 (<0.001)	0.079 (0.307)	−0.061 (0.470)	−0.02 (0.816)	0.13 (0.067)

^*∗*^Multiple regression model. ^*∗∗*^Model I + group.

**Table 4:** Regression model of the effect of academic year and moral sensitivity on professional behaviour of nursing students with adjustment of gender effect.

	B coefficient (SE)	*p* value
Academic year (last year/first year)	6.94 (2.26)	0.002
Moral sensitivity (≥75/<75)	−12.5 (5.05)	0.014
Academic year × moral sensitivity	14.17 (3.2)	<0.001
Gender (male/female)	2.93 (1.95)	0.134

## Data Availability

To maintain the confidentiality of the participants' information, the data of this research are not available.
